# Near-exact non-relativistic ionization energies for many-electron atoms

**DOI:** 10.1098/rsos.211779

**Published:** 2022-03-23

**Authors:** E. O. Jobunga

**Affiliations:** Department of Mathematics and Physics, Technical University of Mombasa, PO Box 90420-80100, Mombasa, Kenya

**Keywords:** electron–electron interaction, all-electron potential, electronic structure theory, nuclear screening parameter, Schrödinger equation, multipole expansion

## Abstract

Electron–electron interactions and correlations form the basis of difficulties encountered in the theoretical solution of problems dealing with multi-electron systems. Accurate treatment of the electron–electron problem is likely to unravel some nice physical properties of matter embedded in the interaction. In an effort to tackle this many-body problem, a symmetry-dependent all-electron potential generalized for an *n*-electron atom is suggested in this study. The symmetry dependence in the proposed potential hinges on an empirically determined angular momentum-dependent partitioning fraction for the electron–electron interaction. With the potential, all atoms are treated in the same way regardless of whether they are open or closed shell using their system specific information. The non-relativistic ground-state ionization potentials for atoms with up to 103 electrons generated using the all-electron potential are in reasonable agreement with the existing experimental and theoretical data. The effects of higher-order non-relativistic interactions as well as the finite nuclear mass of the atoms are also analysed.

## 1. Introduction

The theory of quantum many-body systems is an effective theoretical structure and solvable approach of understanding the collective behaviour of the interacting many-particle systems [1]. The solution of the many-electron problem is important because electrons determine the physical properties of materials and molecules. Many-body physics is heavily applicable in condensed matter, Bose–Einstein condensation (BEC) and superfluidity, quantum chemistry, atomic, molecular, nuclear physics, as well as quantum chromodynamics.

Electron correlation energy, among the interacting many-body particles, is defined as the difference between the exact non-relativistic energy eigenvalue of the electronic Schrödinger equation and the energy of the single configuration state function (CSF) approximation, commonly called the Hartree–Fock energy [2].

Accurate description of electron–electron interaction remains a major challenge in atomic structure calculations [2]. To meet this challenge, a number of different methods have been developed such as the many-body perturbation theory (MBPT) [3], configuration interaction (CI) [4], density functional theory (DFT) [5], coupled cluster theories and different kinds of variational methods [6]. Hylleraas-type calculations [7] are an example of the variational methods in which the interelectronic distance *r*_12_ is employed explicitly in the construction of the wave function resulting in the most accurate eigenvalues, although computationally expensive.

A pseudopotential, or an optimized potential, is an effective potential used as an approximation for the simplified description of complex atoms, molecules and other quantum systems. The use of pseudopotentials was first introduced by Fermi [8]. Hellmann [9] subsequently developed a pseudopotential model for atoms which has been extensively used in atomic scattering [10]. The use of pseudopotential method in the many-body problems is computationally less expensive and has the potential of revealing the underlying processes in the interaction dynamics.

In this work, a central screening potential in an independent-particle model introduced in our previous papers [11–14], based on an alternative multipole expansion of the electron–electron interaction [15], is extended to incorporate the expected symmetry dependence of the electron–electron interaction in the Hamiltonian for an *n*-electron atom. The generalized all-electron potential developed in this work is then used to evaluate the ground-state ionization potentials of atoms with up to 103 electrons.

Atomic systems have been chosen to test the validity and efficiency of the present method in predicting experimental data. The atomic systems are not only useful as a playground for testing physical approximations and numerical algorithms but also provide a basis for understanding complex systems like molecules and condensed matter. Several theoretical studies have been undertaken on atomic systems [16–19] with DFT methods widely adopted. Even though DFT method with local spin density approximation (LSDA) and generalized gradient approximation (GGA) functionals have been used to generate total and ionization energies which are in good agreement with experimental results for up to 86 electrons [17,18], further improvement in the description of atomic systems focusing on non-spherical calculations and on advanced non-local correlation functional is recommended [17].

The symmetry-dependent all-electron potential suggested in this study naturally includes the non-spherical terms of the multipole series expansion of the electron–electron interaction. In the present method, we achieve total separability of the Hamiltonian of the many-electron atom and hence the calculations can be considered to yield near-exact non-relativistic eigenvalues. Our ionization potential results are compared with reported literature data, and with results generated using our previously developed potential which was based on the classical partitioning of the electron–electron interaction [13]. Besides the ionization potentials, we have also included analytically computed partitioning fractions, electron screening parameters, effective nuclear charge, and the ratio of the effective nuclear charge to the unscreened nuclear charge for selected atoms. The information from these additional parameters could be useful in explaining the quantum observables in atomic, molecular and optical physics. Unless explicitly stated, atomic units are used in this study.

## 2. Theory

The infinite nuclear mass non-relativistic Hamiltonian (in atomic units) of an *n*-electron system with a nuclear charge *Z* is given by

2.1 $$H=\sum_{i=1}^{n}\left[\frac{\;p_{i}^{2}}{2}-\frac{Z}{r_{i}}+\sum_{\;j\neq i}^{n-1}\frac{1}{\left|r_{i}-r_{j}\right|}\right],$$

where the first term on the right corresponds to the kinetic energy of the *i*th-electron, the second term corresponds to the interaction of the *i*th-electron with the nuclear charge, and the last term in the summation corresponds to the interaction between the *i*th- and *j*th-electron. The second and the last terms form the potential energy function of a bound *n*-electron system.

In our previous work [15], the binomial expansion and consequently, the multipole expansion of *x*^*k*^,

2.2 $$x^{k}=\sum_{l=\mathrm{even}\;\mathrm{or}\;\mathrm{odd}}^{k}\frac{k!\;(2l+1)}{(k-l)!!(k+l+1)!!}P_{l}(x),$$

where *l* ≤ *k* are both either even or odd non-negative integers and *P*_*l*_(*x*) are the Legender polynomials of order *l*, are used to expand the Coulomb repulsion term. It was shown that the electron–electron interaction analytically simplifies to [15]

2.3 $$\begin{array}{rl} \frac{1}{|r_{i}-r_{j}|} & ={\left(r_{i}^{2}-2r_{i}r_{j}x+r_{j}^{2}\right)}^{-1/2}=\sum_{k=0}^{\infty }\left(\begin{array}{c} -\frac{1}{2}\\ k \end{array}\right){\left(r_{i}^{2}+r_{j}^{2}\right)}^{-(1/2)-k}\;{\left(-2r_{i}\;r_{j}\;x\right)}^{k}\\ & =\frac{4\pi }{\sqrt{r_{i}^{2}+r_{j}^{2}}}\sum_{l=0}^{\infty }\sum_{m=-l}^{+l}{\tilde{\;j}}_{l}(r_{i},r_{j})Y_{l}^{m*}(\hat{r_{i}})Y_{l}^{m}(\hat{r_{j}}), \end{array}$$

where *x* = cos*θ*, *θ* is the angle between vectors ***r***_*i*_ and ***r***_*j*_, the unit vector $\hat{r}$ specifies the angular coordinates of vector ***r***, $Y_{l}^{m}$ are the spherical harmonics which have a correspondence relation with the Legendre polynomials, and

2.4 $${\tilde{\;j}}_{l}(r_{i},r_{j})=\sum_{k=l,l+2,\ldots }^{\infty }\frac{(2k-1)!!}{(k-l)!!\;(k+l+1)!!}{\left(\frac{r_{i}r_{j}}{r_{i}^{2}+r_{j}^{2}}\right)}^{k}$$

are spherical Bessel-like functions. In the independent-particle approximation method, the potential function can be given by

2.5 $$\begin{array}{rl} V(r_{i},r_{j}) & =-\frac{Z}{r_{i}}+\sum_{\;j\neq i}^{n-1}\gamma_{l_{i}}\frac{1}{|r_{i}-r_{j}|}\\ & =-\frac{Z}{r_{i}}+\sum_{\;j\neq i}^{n-1}\gamma_{l_{i}}\frac{4\pi }{\sqrt{r_{i}^{2}+r_{j}^{2}}}\sum_{l,m}{\tilde{\;j}}_{l}(r_{i},r_{j})Y_{l}^{m*}(\hat{r_{i}})Y_{l}^{m}(\hat{r_{j}}), \end{array}$$

for the *i*th-electron of the many-electron atomic system. The coefficient $\gamma_{l_{i}}$ defines the ratio for partitioning the electron–electron interaction energy. Conventionally, factor 1/2 which assumes equal sharing of the Coulomb repulsion energy between the interacting electrons is usually preferred. The interaction potential *V*(*r*_*i*_, *r*_*j*_) where the partitioning fraction is 1/2 can be completely separated by minimizing it with respect to the spatial co-ordinates [13,20].

To obtain reasonable energy eigenvalues for the many-electron atoms, the radially dependent classical partitioning fraction,

2.6 $$\gamma_{i}(r_{i},r_{j})=\frac{r_{i}^{2}}{r_{i}^{2}+r_{j}^{2}},$$

was suggested in [12]. Unlike equal partitioning of the electron–electron interaction, the challenge with the spatially dependent partitioning fraction is that it does not lead to a complete separability of the single-electron potential. This introduces some uncertainties in the calculated energy eigenvalues. To address the challenges associated with the radially dependent partitioning fraction, a symmetry-dependent partitioning fraction [11,14] which depends, not on the radial coordinates, but on the local orbital angular momentum value (*l*_*i*_) for the *i*th state of the system was suggested for two-electron systems. We employed an empirical process [11] to obtain the symmetry-dependent partitioning fractions used in the study.

In this paper, we extend the use of the symmetry-dependent partitioning fractions to derive the single-electron potential for a generalized *n*-electron atom. The potentials developed in our previous paper [13] and other related literature data are used in validating and calibrating the partitioning fractions. Indeed, the success of the equal sharing of the electron–electron interaction potential for spherically symmetric cases observed in [13] already hinted to a possibility of existence of such a symmetry-dependent partitioning fraction. This hint was successfully utilized in coming up with a symmetry-dependent partitioning fraction for helium atom [11,14].

In the empirical determination of the desired partitioning, it is argued that the two interacting electrons share the electron–electron interaction energy, not on equal basis, but with the sharing fractions determined by the proportion of each of their intrinsic energies [11]. The interacting electrons are assumed to be exhibiting quantum harmonic oscillations, with each of their intrinsic energies given by

2.7 $$\epsilon_{i}=\left[{\tilde{l}}_{i}+\frac{1}{2}\right]\hbar \omega ,$$

where ${\tilde{l}}_{i}$ is a discrete quantum number corresponding to the orbital angular momentum of the harmonic oscillator, ℏ is Planck’s constant divided by 2*π*, and *ω* is the fundamental angular frequency of oscillations of the electrons. Consequently, the partitioning fraction corresponding to the *i*th-electron becomes

2.8 $$\gamma_{l_{i}}=\frac{\epsilon_{i}}{\epsilon_{i}+\epsilon_{j}},$$

which simplifies to the form given by equation (2.9). The empirical task is then reduced to the determination of the explicit forms of *δ*_*i*_ and δ_j_ in equation (2.9).

In our working, we established that the symmetry-dependent partitioning fraction is system specific and largely takes a general form which depends on the symmetry of the valence and active electrons. For valence electrons with an orbital angular momentum value *l*_*v*_ and an orbital angular momentum *l*_*i*_ for the *i*th electron, the partitioning fraction $\gamma_{l_{i}}$ is empirically determined [14] to be

2.9 $$\gamma_{l_{i}}=\frac{1+\delta_{i}}{2+\delta_{i}+\delta_{j}},$$

where the parameters are given by

2.10 $$\delta_{i}=\left\{ \begin{array}{cc} 0\; & \text{if }l_{i}=0\\ {l_{i}}_{\;\sqrt{l_{i}}} & \text{if }l_{i}\neq 0 \end{array}\right.$$

and

2.11 $$\delta_{\;j}=\frac{l_{v}}{2^{\left(2l_{v}-1\right)}},$$

respectively. The non-local parameter *δ*_*j*_ in equation (2.9) is accounted for by the valent subshell with *l*_*v*_ being the orbital angular momentum quantum number of the valent electron for the atom in its ground-state configuration.

With the suggested symmetry-dependent partitioning fraction, the lowest-order interaction potential

2.12 $$\begin{array}{rl} V_{0}^{0}(r_{i},r_{j}) & =-\frac{Z}{r_{i}}+\sum_{\;j\neq i}^{n-1}\gamma_{l_{i}}\;\frac{1}{\sqrt{r_{i}^{2}+r_{j}^{2}}}\\ & =\sum_{\;j\neq i}^{n-1}\left[-\frac{Z}{(n-1)r_{i}}+\gamma_{l_{i}}\frac{1}{\sqrt{r_{i}^{2}+r_{j}^{2}}}\right], \end{array}$$

for the *i*th electron obtained by setting *l* = *k* = 0 in the approximation of the multipole electron-electron interaction used in equation (2.5), can be minimized by differentiating it with respect to *r*_*i*_

2.13 $$\frac{\partial }{\partial r_{i}}V_{0}^{0}(r_{i},r_{j})=0,$$

and equating the derivative to zero. Physically, this corresponds to enforcing an equilibrium condition requiring the net force acting on the *i*th electron to vanish. This leads to the separation

2.14 $$\frac{1}{\sqrt{r_{i}^{2}+r_{j}^{2}}}=\frac{{\;}^{3}\sqrt{Z/[(n-1)\gamma_{l_{i}}]}}{r_{i}},$$

of the entangled coordinates within the lowest-order spherical approximation.

Equation (2.14) and a further mean-field approximation of the spherical Bessel-like functions, given by equation (2.4), are used to simplify equation (2.5). This yields the symmetry-dependent single-electron multipole potential [14]

2.15 $$V(r_{i})=\sum_{l=0}^{l_{\max}\to \infty }\sum_{m=-l}^{l}V_{l}{\;}^{m}(r_{i}),$$

for the *n*-electron system where

2.16 $$\begin{array}{rl} V_{0}^{0}(r_{i}) & =-\frac{\left[Z-(n-1)\gamma_{l_{i}}\langle {\tilde{\;j}}_{0}(r_{i},r_{j})\rangle {\;}^{3}\sqrt{Z/\left[(n-1)\gamma_{l_{i}}\right]}\right]}{r_{i}},\\ \mathrm{and}\;\;\;\;V_{l\neq 0}^{m}(r_{i}) & =4\pi (n-1)\gamma_{l_{i}}\;A_{l}^{m}\left\langle {\tilde{\;j}}_{l}(r_{i},r_{j})\right\rangle \frac{{\;}^{3}\sqrt{Z/\left[(n-1)\gamma_{l_{i}}\right]}}{r_{i}}\\ & \;\times \frac{R_{n_{j},l_{j}}(r_{i})}{R_{n_{i},l_{i}}(r_{i})}(R_{n_{i},l_{i}}(r_{j})|R_{n_{j},l_{j}}(r_{j})). \end{array}\}$$

The higher-order multipole potential terms, $V_{l\neq 0}^{m}(r_{i})$,

2.17 $$\begin{array}{rl} V_{l\neq 0}^{m}(r_{i}) & =4\pi (n-1)\gamma_{l_{i}}A_{l}^{m}\langle {\tilde{\;j}}_{l}(r_{i},r_{j})\rangle \frac{{\;}^{3}\sqrt{Z/\left[(n-1)\gamma_{l_{i}}\right]}}{r_{i}}|N_{n_{j},l_{j}}{|}^{2}\\ & \;\times \frac{r_{i}^{l_{j}}}{r_{i}^{l_{i}}}\exp\left[-\left(\frac{l_{i}-l_{j}}{\left(l_{i}+1)(l_{j}+1\right)}\right)Zr_{i}\right]\\ & \;\times \int_{0}^{\infty }r_{j}^{2+l_{i}+l_{j}}\exp\left[-\left(\frac{2+l_{i}+l_{j}}{\left(l_{i}+1)(l_{j}+1\right)}\right)Zr_{j}\right]\;dr_{j}\delta_{l_{i},l_{j}+l}\delta_{m_{i},m_{j}+m}\\ & =4\pi (n-1)\gamma_{l_{i}}A_{l}^{m}\langle {\tilde{\;j}}_{l}(r_{i},r_{j})\rangle \frac{{\;}^{3}\sqrt{Z/\left[(n-1)\gamma_{l_{i}}\right]}}{r_{i}^{l+1}}|N_{n_{j},l_{j}}{|}^{2}\\ & \;\times \exp\left[-\left(\frac{l}{\left(l_{i}+1)(l_{i}-l+1\right)}\right)Zr_{i}\right]\\ & \;\times \frac{(2l_{i}-l+2)!{\left[(l_{i}+1)(l_{i}-l+1)\right]}^{\left(2l_{i}-l+3\right)}}{{\left[(2l_{i}-l+2)Z\right]}^{\left(2l_{i}-l+3\right)}}\delta_{l_{i},l_{j}+l}\delta_{m_{i},m_{j}+m}, \end{array}$$

emanating from the inner integral of the double integration (in space coordinates) are further evaluated using Laplace transform method and hydrogenic radial wave functions

2.18 $$R_{n^{\prime }l^{\prime }}(r)=N_{n^{\prime },l^{\prime }}r^{l^{\prime }}\exp\left(-\frac{Zr}{l^{\prime }+1}\right),$$

as our trial functions. In equation (2.18), *N*_*n*′,*l*′_ are the normalization factors, *n*′ = *l*′ + 1and *l*′ are the principal and the orbital angular momentum quantum numbers of the hydrogenic trial functions respectively. In equation (2.17), we have imposed the conditions *l*_*i*_ = *l*_*j*_ + *l* and *m*_*i*_ = *m*_*j*_ + *m* in the triangular relations of the angular momentum algebra. The angular factors $A_{l}^{m}$ arise from the angular integral and can be evaluated with the help of the Wigner-3*j* symbols as

2.19 $$\begin{array}{rl} A_{l}^{m} & =\int Y_{l_{j}}^{m_{j}}Y_{l}^{m*}Y_{l_{i}}^{m_{i}}\;d\Omega_{i}\times \int Y_{l_{i}}^{m_{i}}Y_{l}^{m}Y_{l_{j}}^{m_{j}}\;d\Omega_{j}\\ & ={\left(-1\right)}^{m}\frac{\left(2l_{i}+1)(2l+1)(2l_{j}+1\right)}{4\pi }{\left(\begin{array}{ccc} l_{i} & l & l_{j}\\ 0 & 0 & 0 \end{array}\right)}^{2}{\left(\begin{array}{ccc} l_{i} & l & l_{j}\\ m_{i} & m & m_{j} \end{array}\right)}^{2}. \end{array}$$

In principle, the triangular relations allow *l*_*i*_ = *l*_*j*_ ± *l* but the inclusion of *l*_*i*_ = *l*_*j*_ − *l* leads to divergences in the Hamiltonian. This can be explained that the exchange of quantum numbers between the electrons is mediated by the operator and can go only in one direction such that *l*_*i*_ = *l*_*j*_ + *l*.

Within the mean-field approximation, the multipole potential terms in equations (2.16) and (2.17) can compactly be expressed as

2.20 $$\begin{array}{rl} V_{0}^{0}(r_{i}) & =-\frac{\left[Z-(n-1)\gamma_{l_{i}}B_{0}^{0}(Z){\;}^{3}\sqrt{Z/\left[(n-1)\gamma_{l_{i}}\right]}\right]}{r_{i}}\\ \mathrm{and}\;\;\;\;V_{l\neq 0}^{m}(r_{i}) & =(n-1)\gamma_{l_{i}}B_{l}^{m}(Z){\;}^{3}\sqrt{\frac{Z}{(n-1)\gamma_{l_{i}}}}\\ & \;\times \frac{\exp[-(lZr/\left(l_{i}+1)(l_{i}-l+1\right))]}{r_{i}^{l+1}}\delta_{l_{i},l_{j}+l}\delta_{m_{i},m_{j}+m}, \end{array}\}$$

with the coefficient

2.21 $$\begin{array}{rl} B_{l}^{m}(Z) & =\frac{(2+2l_{i}-l)!}{(2l_{i}-2l+2)!}{\left(\frac{2Z}{l_{i}-l+1}\right)}^{\left(2l_{i}-2l+3\right)}\\ & \;\times {\left[\frac{\left(l_{i}+1)(l_{i}-l+1\right)}{(2+2l_{i}-l)Z}\right]}^{2l_{i}-l+3}A_{l}^{m}\langle {\tilde{\;j}}_{l}(r_{i},r_{j})\rangle . \end{array}$$

The expectation values of the spherical Bessel-like functions,

2.22 $$\langle {\tilde{\;j}}_{l}(r_{i},r_{j})\rangle ={\left(\frac{1}{4\pi \sqrt{2}}\right)}^{l}\sum_{k=0,2,\ldots }^{\infty }\frac{(2l+2k-1)!!}{k!!(2l+k+1)!!}{\left(\frac{1}{4\pi \sqrt{2}}\right)}^{k},$$

are approximated using a mean value of its argument, that is, the square root of the argument’s peak value per solid angle. The higher-order multipole potentials $V_{l}^{m}(r_{i})$ can then be added perturbatively, as per equation (2.15), to increase the accuracy of the single-electron potential.

The summation of all orders of the multipole potential terms in equations (2.15) and (2.20) simplify to a fully analytical single-electron potential function given by [21–24]

2.23 $$V(r_{i})=-\frac{\left(Z-\sigma_{l_{i}}^{n}\right)}{r_{i}},$$

where the electron screening parameter, $\sigma_{l_{i}}^{n}$, dependent on the number of electrons *n*, orbital angular momentum *l*_*i*_, and the angular momentum of the valent electrons in ground state *l*_*v*_ , is evaluated as

2.24 $$\begin{array}{rl} \sigma_{l_{i}}^{n} & =(n-1)\gamma_{l_{i}}{\;}^{3}\sqrt{\frac{Z}{(n-1)\gamma_{l_{i}}}}\sum_{l=0}^{l_{\max}\to \infty }\sum_{m=-l}^{+l}B_{l}^{m}(Z)\\ & \;\times \frac{\exp[-(lZr/\left(l_{i}+1)(l_{i}-l+1\right))]}{r_{i}^{l}}\delta_{l_{i},l_{j}+l}\delta_{m_{i},m_{j}+m}. \end{array}$$

Within the spherical approximation, the electron screening parameter simplifies to

2.25 $$\sigma_{l_{i}}^{n}\approx (n-1)\gamma_{l_{i}}\;\langle {\tilde{\;j}}_{0}\rangle {\;}^{3}\sqrt{\frac{Z}{(n-1)\gamma_{l_{i}}}},$$

with the expectation value $\langle {\tilde{\;j}}_{0}\rangle \approx 1.001591982$ evaluated using equation (2.22).

Using the suggested symmetry-dependent all-electron potential given by equation (2.23), the one-electron Hamiltonian,

2.26 $$h_{\infty }(r_{i})=\frac{\;p_{i}^{2}}{2}+V(r_{i}),$$

without the finite nuclear mass correction is defined. With the finite nuclear mass correction, the Hamiltonian becomes

2.27 $$h(r_{i})=h_{\infty }(r_{i})-\frac{1}{M_{ion}}h_{\infty }(r_{i}),$$

where 1/*M*_ion_ is the electron-atomic nuclear mass ratio.

The eigenvalue $\epsilon_{\alpha_{i}}$ corresponding to a state with the quantum numbers *α*_*i*_=$\left\{n_{i},l_{i},m_{i}\right\}$ for an *n*-electron atom can then be generalized as

2.28 $$\epsilon_{\alpha_{i}}=\frac{m}{n}\langle \phi_{\alpha_{i}}|h(r_{i})|\phi_{\alpha_{i}}\rangle ,$$

where *m*/*n* refers to the proportion of non-vanishing integrals out of all the possible permutations. For lithium, *m*/*n* = 2/3 as was already shown in our previous paper [13]. In principle, the integer *m* can be determined from the spin-allowed ground-state configuration of the atom, but constrained further by other symmetry considerations. We have provided electronic supplementary material [25] showing how the various values of *m*/*n* have been determined for up to *n* = 7. For atoms with a higher number of electrons, we have used an intuitive reasoning based on the arrangement of the systems in the periodic table to determine the values of *m*/*n* [25]. A complete understanding of the symmetry relations for such large systems is, however, still necessary. The ratio 1 : *m* − 1 corresponds to the contribution of the direct and exchange integrals involved in the evaluation of the energy of the system respectively. This shows that, apart from helium and alkalis where the exchange integral has an equal weight with the direct integral, the exchange contribution is greater in all the other atoms. Apparently, *m* has a maximum value of 5 for noble gases regardless of the number of electrons present in the atom.

For comparison purposes, we have also included results calculated using our previously derived lowest-order non-relativistic central potential [13]

2.29 $$V(r_{i})=-\frac{Z}{r_{i}}+(n-1)\frac{{\left[Z\;f\;(r_{i},r_{j})/2(n-1)\right]}^{3/5}}{r_{i}},$$

where the expectation value,

2.30 $$\langle f\;{\left(r_{i},r_{j}\right)}^{3/5}\rangle \approx 1-\left[\frac{27}{25}+\frac{3}{5}Zr_{i}-\frac{6}{125\;Zr_{i}}\right]\exp(-2Zr_{i}),$$

is approximately optimized by evaluating the integral using a trial function for hydrogenic system in the 1*s* state.

Within the spherical approximation, the infinite nuclear mass energy eigenvalue, corresponding to the principal quantum number *n*_*i*_, for the *i*th electron can be computed analytically using the relation

2.31 $$\epsilon_{\alpha_{i}}=-\frac{m}{n}\times \frac{Z_{\mathrm{eff}}^{2}}{2{n_{i}}^{2}},$$

where the screened effective nuclear charge *Z*_eff_ = *Z* − *σ*_0_^n^ is evaluated using equation (2.25). This relation can be seen to be similar to the one for hydrogen-like eigenvalues, but scaled with a system-dependent factor *m*/*n*.

## 3. Results and discussions

We have developed a symmetry-dependent all-electron potential for an *n*-electron system defined by equations (2.15) and (2.20). The potentials are used to calculate the ground-state ionization potentials for *n*-electron atoms as shown in tables 2 and 3 with 2 ≤ *n* ≤ 103. Our results are compared with the results of the central potential given by equation (2.29), density functional theory exchange correlation (DFT-XC) calculations [17], and experimental reference data [26]. In generating our results, a B-spline radial box of 600 B-splines, maximum radius *r*_max_ = 200, order *k* = 10, and a nonlinear knot sequence is used. Our results can also be evaluated analytically using equation (2.31), if the suggested all-electron potential is restricted to the spherical terms only, by determining the nuclear screening parameters and the effective nuclear charge.

In table 1, the partitioning fractions (P.F.), electron screening parameters (*σ*), effective nuclear charge (*Z*_eff_), infinite nuclear mass non-relativistic ionization potentials (*ε*_*α*_) in eV, and the ratio of the effective nuclear charge to the unscreened nuclear charge (*Z*_eff_/*Z*) are analytically computed, with a desk calculator, within the spherical approximation for some atoms using equations (2.9), (2.25), and (2.31), respectively. The ionization potentials are obtained using Koopman’s theorem [27] except for helium atom, which is determined by multiplying the energy eigenvalue by 4 and then subtracting −2.0, the ground-state energy for helium ion, to get the binding energy for the 1*s* electron. The agreement with the numerically evaluated results presented in tables 2 and 3 is excellent.

**Table 1.** Analytically computed partitioning fractions (P.F.), electron screening parameters (*σ*), effective nuclear charge (*Z*_eff_), infinite nuclear mass non-relativistic ionization potentials (*ε*_*α*_) in eV, and the ratio of the effective nuclear charge to the unscreened nuclear charge (*Z*_eff_/*Z*) within the spherical approximation for some atoms. Equations (2.9), (2.25) and (2.31), respectively, have been used in the computations.

*n*/*Z*	atom	state	*m*/*n*	P.F.	*σ*	*Z*_eff_	*ε*_*α*_ (eV)	*Z*_eff_/*Z*
2	He	1s	2/2	0.5000	0.7950	1.205	24.60	0.6025
3	Li	2s	2/3	0.5000	1.4445	1.5555	5.48	0.5185
4	Be	2s	3/4	0.5000	2.0834	1.9166	9.37	0.4791
5	B	2p	3/5	0.5714	2.9718	2.0282	8.39	0.4056
6	C	2p	4/6	0.5714	3.6645	2.3355	12.36	0.3892
7	N	2p	4/7	0.5714	4.3563	2.6437	13.58	0.3777
8	O	2p	4/8	0.5714	5.0475	2.9525	14.82	0.3691
9	F	2p	4/9	0.5714	5.7384	3.2616	16.08	0.3624
10	Ne	2p	5/10	0.5714	6.4290	3.571	21.68	0.3571
11	Na	3s	2/11	0.5000	6.5133	4.4867	5.53	0.4079
12	Mg	3s	3/12	0.5000	7.1448	4.8552	8.90	0.4046
13	Al	3p	3/13	0.5714	8.5000	4.5000	7.06	0.3462
14	Si	3p	4/14	0.5714	9.1901	4.8099	9.99	0.3436
15	P	3p	4/15	0.5714	9.8802	5.1198	10.56	0.3413
16	S	3p	4/16	0.5714	10.5702	5.4298	11.14	0.3394
17	Cl	3p	4/17	0.5714	11.2602	5.7398	11.71	0.3376
18	Ar	3p	5/18	0.5714	11.9502	6.0498	15.36	0.3361
19	K	4s	2/19	0.5000	11.5639	7.4361	4.94	0.3914
20	Ca	4s	3/20	0.5000	12.1950	7.8050	7.77	0.3903
21	Sc	3d	3/21	0.6589	15.4169	5.5831	6.73	0.2659
39	Y	4d	3/39	0.6589	29.0702	9.9298	6.44	0.2546
57	La	4f	3/57	0.6907	44.0860	12.9140	7.46	0.2266
89	Ac	5f	3/89	0.6907	69.1304	19.8696	7.24	0.2233

**Table 2.** Some numerically calculated non-relativistic ionization potentials (in eV) for 2 ≤ *n* ≤ 54-electron atoms versus the reference values from DFT-XC (LSDA and GGA) calculations [17] and experiment [26]. The present *V*_cen_ results are evaluated using equation (2.29) and $V_{0,0}^{0}$ are evaluated using the lowest-order symmetry-dependent non-relativistic potential given by equation (2.20) with *j*_0_(*r*_*i*_, *r*_*j*_) ≈ 1. The present *V*_0_ and *V*_*h*_ are the results evaluated using the symmetry-dependent all-electron multipole potential given by equations (2.15) and (2.20) by fixing *l*_max_ = 0 and *l*_max_ = 4 respectively. The present *V*_fnm_ results are obtained by including the finite nuclear mass scaling corrections, using equation (2.27), on the multipole *V*_*h*_ data. The results presented are truncated to 2 d.p. The deviations Δɛ are determined by subtracting the experimental results from our *V*_fnm_ results.

*n*	atom	state	*m*/*n*	*V*_cen_	$V_{0,0}^{0}$	*V*_0_	*V*_*h*_	*V*_fnm_	LSDA	GGA	Exp. (eV)	Δɛ
2	He	1s	2/2	35.21	24.77	24.60	24.60	24.59	24.29	24.46	24.58	+0.01
3	Li	2s	2/3	4.97	5.50	5.48	5.48	5.48	5.47	5.58	5.39	+0.09
4	Be	2s	3/4	8.91	9.40	9.37	9.37	9.36	9.02	8.99	9.32	+0.04
5	B	2p	3/5	8.08	8.43	8.39	8.35	8.35	8.57	8.48	8.29	+0.06
6	C	2p	4/6	12.29	12.42	12.36	12.31	12.30	11.76	11.67	11.26	+1.04
7	N	2p	4/7	13.83	13.65	13.58	13.52	13.51	14.99	14.91	14.53	−1.02
8	O	2p	4/8	15.37	14.90	14.82	14.76	14.75	13.89	13.67	13.61	+1.14
9	F	2p	4/9	16.93	16.16	16.07	16.01	16.00	18.05	17.84	17.42	−1.42
10	Ne	2p	5/10	23.12	21.80	21.68	21.59	21.58	22.17	21.98	21.56	+0.02
11	Na	3s	2/11	5.30	5.55	5.53	5.53	5.52	5.36	5.35	5.13	+0.39
12	Mg	3s	3/12	8.59	8.95	8.90	8.90	8.90	7.60	7.71	7.64	+1.26
13	Al	3p	3/13	7.72	7.10	7.06	7.04	7.03	5.99	5.96	5.98	+1.05
14	Si	3p	4/14	10.99	10.05	9.99	9.96	9.95	8.27	8.27	8.15	+1.80
15	P	3p	4/15	11.69	10.63	10.56	10.53	10.52	10.53	10.55	10.48	+0.04
16	S	3p	4/16	12.39	11.20	11.14	11.10	11.09	10.53	10.23	10.36	+0.73
17	Cl	3p	4/17	13.09	11.79	11.71	11.68	11.67	13.24	13.05	12.96	−1.29
18	Ar	3p	5/18	17.24	15.46	15.36	15.32	15.30	15.93	15.80	15.75	−0.45
19	K	4s	2/19	4.65	4.97	4.94	4.94	4.94	4.52	4.44	4.34	+0.60
20	Ca	4s	3/20	7.31	7.80	7.77	7.77	7.75	6.20	6.06	6.11	+1.64
21	Sc	3d	3/21	11.86	6.79	6.73	6.71	6.70	6.54	6.37	6.56	+0.14
22	Ti	3d	3/22	12.39	7.05	6.99	6.97	6.96	6.71	6.54	6.82	+0.14
23	V	3d	3/23	12.91	7.32	7.25	7.24	7.22	7.14	6.96	6.74	+0.48
24	Cr	3d	3/24	13.43	7.58	7.51	7.50	7.48	7.45	7.25	6.76	+0.72
25	Mn	3d	3/25	13.96	7.85	7.78	7.76	7.75	7.48	7.15	7.43	+0.32
26	Fe	3d	3/26	14.48	8.11	8.04	8.02	8.01	8.20	7.84	7.90	+0.11
27	Co	3d	3/27	15.01	8.38	8.30	8.28	8.27	8.10	7.89	7.88	+0.39
28	Ni	3d	3/28	15.53	8.64	8.57	8.55	8.53	8.23	8.01	7.63	+0.90
29	Cu	3d	3/29	16.06	8.91	8.83	8.81	8.79	8.38	8.14	7.72	+1.07
30	Zn	3d	3/30	16.58	9.17	9.09	9.07	9.05	9.68	9.37	9.39	−0.34
31	Ga	4p	3/31	9.66	8.41	8.36	8.34	8.32	6.05	5.90	5.99	+2.33
32	Ge	4p	4/32	13.27	11.55	11.47	11.45	11.42	8.06	7.97	7.89	+3.53
33	As	4p	4/33	13.67	11.88	11.80	11.78	11.75	9.97	9.93	9.78	+1.97
34	Se	4p	4/34	14.06	12.21	12.13	12.10	12.07	9.92	9.54	9.75	+2.32
35	Br	4p	4/35	14.46	12.54	12.45	12.43	12.40	12.12	11.87	11.81	+0.59
36	Kr	4p	5/36	18.56	16.08	15.98	15.94	15.90	14.26	14.09	13.99	+1.91
37	Rb	5s	2/37	5.42	5.85	5.82	5.82	5.81	4.32	4.21	4.17	+1.64
38	Sr	5s	3/38	8.35	9.01	8.96	8.96	8.94	5.77	5.61	5.69	+3.25
39	Y	4d	3/39	11.98	6.51	6.45	6.43	6.42	6.24	6.02	6.21	+0.21
40	Zr	4d	3/40	12.27	6.66	6.59	6.58	6.56	6.68	6.48	6.63	−0.07
41	Nb	4d	3/41	12.57	6.81	6.74	6.73	6.71	7.03	6.80	6.75	−0.04
42	Mo	4d	3/42	12.86	6.96	6.89	6.88	6.86	7.29	7.04	7.09	−0.23
43	Tc	4d	3/43	13.16	7.11	7.04	7.03	7.01	7.45	7.21	7.11	−0.10
44	Ru	4d	3/44	13.46	7.26	7.19	7.17	7.15	7.54	7.29	7.36	−0.21
45	Rh	4d	3/45	13.75	7.41	7.34	7.32	7.30	8.29	7.97	7.45	−0.15
46	Pd	4d	3/46	14.05	7.56	7.49	7.47	7.45	9.37	9.08	8.33	−0.88
47	Ag	4d	3/47	14.34	7.71	7.63	7.62	7.60	7.66	7.36	7.57	+0.03
48	Cd	4d	3/48	14.64	7.86	7.78	7.77	7.74	8.85	8.50	8.99	−1.25
49	In	5p	3/49	9.58	8.23	8.17	8.16	8.13	5.77	5.58	5.78	+2.35
50	Sn	5p	4/50	13.03	11.18	11.11	11.09	11.05	7.48	7.36	7.34	+3.71
51	Sb	5p	4/51	13.28	11.39	11.32	11.30	11.26	9.08	9.01	8.60	+2.66
52	Te	5p	4/52	13.54	11.60	11.53	11.51	11.47	9.06	8.69	9.00	+2.47
53	I	5p	4/53	13.79	11.82	11.74	11.72	11.67	10.85	10.59	10.45	+1.22
54	Xe	5p	5/54	17.55	15.03	14.93	14.91	14.85	12.56	12.38	12.12	+2.73

**Table 3.** Same as table 2 but for 55 ≤ *n* ≤ 103-electron atoms.

*n*	atom	state	*m*/*n*	*V*_cen_	$V_{0,0}^{0}$	*V*_0_	*V*_*h*_	*V*_fnm_	LSDA	GGA	Exp.(eV)	Δ*ε*
55	Cs	6s	2/55	5.42	5.93	5.89	5.89	5.87	3.94	3.83	3.89	+1.98
56	Ba	6s	3/56	8.28	9.05	9.00	9.00	8.96	5.16	4.97	5.21	+3.75
57	La	4f	3/57	17.29	7.54	7.46	7.45	7.42	5.31	4.98	5.57	+1.85
58	Ce	4f	3/58	17.59	7.66	7.58	7.57	7.54	5.12	4.96	5.53	+2.01
59	Pr	4f	3/59	17.88	7.78	7.70	7.69	7.66	4.99	4.82	5.47	+2.19
60	Nd	4f	3/60	18.18	7.91	7.82	7.81	7.78	4.88	4.70	5.52	+2.26
61	Pm	4f	3/61	18.47	8.03	7.94	7.93	7.90	4.78	4.60	5.58	+2.32
62	Sm	4f	3/62	18.77	8.15	8.06	8.05	8.02	4.70	4.53	5.64	+2.38
63	Eu	4f	3/63	19.06	8.27	8.18	8.17	8.13	5.51	5.33	5.67	+2.46
64	Gd	4f	3/64	19.36	8.39	8.30	8.29	8.25	5.60	5.42	6.14	+2.11
65	Tb	4f	3/65	19.65	8.51	8.42	8.41	8.37	5.49	5.35	5.86	+2.51
66	Dy	4f	3/66	19.95	8.64	8.54	8.53	8.49	5.40	5.26	5.93	+2.56
67	Ho	4f	3/67	20.24	8.76	8.66	8.65	8.61	5.32	5.17	6.02	+2.59
68	Er	4f	3/68	20.54	8.88	8.78	8.77	8.73	5.26	5.10	6.10	+2.63
69	Tm	4f	3/69	20.84	9.00	8.90	8.89	8.85	5.20	5.07	6.18	+2.67
70	Yb	4f	3/70	21.13	9.12	9.02	9.01	8.97	6.00	5.82	6.25	+2.72
71	Lu	5d	3/71	13.71	7.24	7.17	7.16	7.12	6.39	6.18	5.42	+1.70
72	Hf	5d	3/72	13.90	7.33	7.26	7.25	7.22	6.76	6.59	6.82	+0.40
73	Ta	5d	3/73	14.09	7.43	7.36	7.35	7.31	7.23	7.01	7.54	−0.23
74	W	5d	3/74	14.28	7.53	7.45	7.44	7.40	7.49	7.25	7.86	−0.46
75	Re	5d	3/75	14.47	7.62	7.55	7.54	7.50	7.63	7.15	7.83	−0.33
76	Os	5d	3/76	14.66	7.72	7.64	7.63	7.59	7.69	7.45	8.43	−0.84
77	Ir	5d	3/77	14.84	7.81	7.74	7.73	7.69	8.57	8.24	8.96	−1.27
78	Pt	5d	3/78	15.03	7.91	7.83	7.82	7.78	9.67	9.38	8.95	−1.17
79	Au	5d	3/79	15.22	8.01	7.93	7.92	7.87	7.75	7.45	9.22	−1.35
80	Hg	5d	3/80	15.41	8.10	8.03	8.01	7.97	8.82	8.46	10.43	−2.46
81	Tl	6p	3/81	10.86	9.23	9.16	9.15	9.10	5.67	5.46	6.10	+3.00
82	Pb	6p	4/82	14.66	12.45	12.37	12.35	12.28	7.25	7.12	7.41	+4.87
83	Bi	6p	4/83	14.83	12.60	12.51	12.49	12.42	8.71	8.63	7.28	+5.14
84	Po	6p	4/84	15.01	12.74	12.66	12.64	12.57	8.69	8.32	8.41	+4.16
85	At	6p	4/85	15.18	12.89	12.80	12.79	12.71	10.29	10.02	9.31	+3.40
86	Rn	6p	5/86	19.20	16.30	16.19	16.16	16.07	11.81	11.63	10.74	+5.33
87	Fr	7s	2/87	6.15	6.78	6.75	6.75	6.71			4.07	+2.64
88	Ra	7s	3/88	9.33	10.29	10.24	10.24	10.18			5.27	+4.91
89	Ac	5f	3/89	17.11	7.32	7.24	7.23	7.18			5.38	+1.80
90	Th	5f	3/90	17.30	7.40	7.32	7.30	7.26			6.30	+0.96
91	Pa	5f	3/91	17.49	7.47	7.39	7.38	7.34			5.89	+1.45
92	U	5f	3/92	17.68	7.55	7.47	7.46	7.41			6.19	+1.22
93	Np	5f	3/93	17.87	7.63	7.55	7.54	7.49			6.26	+1.23
94	Pu	5f	3/94	18.06	7.71	7.62	7.61	7.56			6.02	+1.54
95	Am	5f	3/95	18.25	7.79	7.70	7.69	7.64			5.97	+1.67
96	Cm	5f	3/96	18.44	7.86	7.78	7.77	7.72			5.99	+1.73
97	Bk	5f	3/97	18.62	7.94	7.86	7.84	7.79			6.19	+1.60
98	Cf	5f	3/98	18.81	8.02	7.93	7.92	7.87			6.28	+1.59
99	Es	5f	3/99	19.00	8.10	8.01	8.00	7.94			6.36	+1.58
100	Fm	5f	3/100	19.19	8.18	8.09	8.07	8.02			6.50	+1.52
101	Md	5f	3/101	19.38	8.25	8.16	8.15	8.10			6.58	+1.52
102	No	5f	3/102	19.57	8.33	8.24	8.23	8.17			6.62	+1.55
103	Lr	6d	3/103	13.72	7.16	7.09	7.08	7.03			4.96	+2.07

From table 1, we can observe that for a given symmetry, the charge screening effect, as measured using *σ*/*Z* = 1 − *Z*_eff_/*Z* ratio, is a slowly increasing function of nuclear charge for the neutral atoms. It can also be seen that the effect of nuclear screening is strongly dependent on the partitioning fractions (P.F). The higher the partitioning fraction, the higher the nuclear shielding effect, if nuclear charge is held constant. The fraction *σ*/*Z* is lower for *s* states and respectively higher for *p*, *d* and *f* states. Additionally, the variations of the ionization potential in the periodic table can also be attributed to the symmetry scaling factor *m*/*n* in equation (2.28), besides the principal quantum number of the valent electron.

Tables 2 and 3 show some numerically calculated non-relativistic ionization energies for *n*-electron atoms using the present central lowest-order potential *V*_cen_ given by equation (2.29) and the symmetry-dependent all-electron potentials in equations (2.15) and (2.20), evaluated by fixing *j*_0_(*r*_*i*_, *r*_*j*_) ≈ 1 for the lowest-order $V_{0,0}^{0}$ term, and *l*_max_ = 0 and *l*_max_ = 4 for the higher-order monopole *V*_0_ and multipole *V*_*h*_ terms, respectively. We have also included the finite nuclear mass corrections in our higher multipole order *V*_fnm_ results. Our results are compared with the reference theoretical [17] and experimental values [26]. The tables also contain the ground-state valent orbital, the energy deviation (Δ*ε*) from the experimental values as well as the single-electron Hamiltonian scaling factor (*m*/*n*) for each of the atoms, already introduced in equation (2.28), that have been determined [25] and used in the present study. These scaling factors yield information on the relative importance of the exchange integrals, arising from the permutation symmetry, in the evaluation of the energy eigenvalues. They are also useful in explaining the shape of ionization potential curves as a function of the nuclear charge *Z* in the periodic table of atoms.

The ionization energies have been obtained using Koopmans’ theorem [27], except for the ground state of helium atom, which is evaluated as the difference between the total energy of helium atom and the residual ion both in their ground states. The ground-state ionization energies calculated using the present all-electron potential incorporating the multipole terms, without (*V*_*h*_) and with the finite nuclear mass correction (*V*_fnm_), as given by equations (2.15), (2.20), (2.26) and (2.27) are in fair agreement with the reference values. We have also included the results generated using the lowest-order central (*V*_cen_) and symmetry-dependent ($V_{0,0}^{0}$) non-relativistic potential as well as a higher-order monopole spherical (*V*_0_) potential. With these additional potentials, the effects of the dominant lowest-order interaction, the central potential, the symmetry-dependent potential, the higher-order spherical and non-spherical interactions, and of the finite nuclear mass corrections can be investigated.

The central potential (*V*_cen_) given by equation (2.29) and the symmetry-dependent potential ($V_{0,0}^{0}$) interaction terms are both lowest-order non-relativistic interaction terms of the multipole interaction potential, with the difference between them being the nature of the partitioning fraction ($\gamma_{l_{i}}$) used for each case. In one case, a radial partitioning fraction is used, while in another case, a symmetry-dependent partitioning fraction is used in the treatment of the electron–electron interaction terms. The results between the two corresponding potentials compare well qualitatively but the quantitative difference increases with the atomic nuclear charge (*Z*). The discrepancy between the results generated by the lowest-order multipole potentials can be attributed to the approximation involved in the central potential given by equation (2.30). Since the calculations involving the symmetry-dependent partitioning fraction can be considered to be exact, without any approximation used, their values can be considered to be reliable within the non-relativistic regime, subject to the validity of the use of both the partitioning and scaling fractions suggested in this work.

Indeed, if the higher-order interactions and finite nuclear mass corrections are included into the symmetry-dependent single-electron Hamiltonian, there is some slight improvement in the agreement of our results with the experimental values for all the multi-electron atoms. The trend of the ionization energies in the periodic table is correctly predicted by our all-electron potential. The quantitative agreement with experimental values is better for few-electron atoms but decrease with higher *Z*-values. Some major discrepancies are, however, observed between our evaluated results and the experimental results for some of the multi-electron atoms across the *Z* spectrum. For example, our potential is unable to explain why the ionization energy of oxygen atom is lower than that of nitrogen atom. The source of such discrepancies is not quite clear but some can be attributed to the dominant role played by the relativistic effects to some extent, especially for high *Z* atoms. It is of desirable interest to include the effects arising from the relativistic and other higher-order interactions in our future investigation.

**Figure 1.**
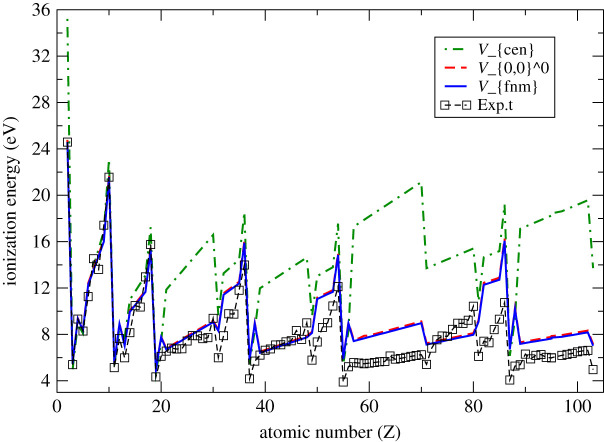
Non-relativistic atomic ionization energies for multi-electron atoms evaluated using various potentials in comparison with experimental results [26].

**Figure 2.**
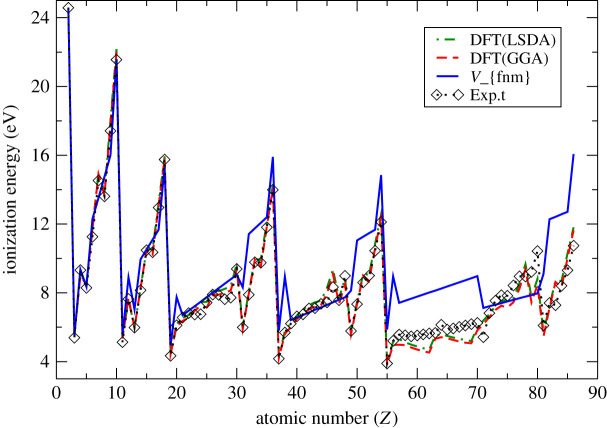
Non-relativistic atomic ionization energies for multi-electron atoms evaluated using our symmetry-dependent higher-order multipole potential, with finite nuclear mass corrections (V_{fnm}), in comparison with DFT-XC (LSDA and GGA) [17] and experimental results [26].

Figures 1 and 2 graphically represent the results contained in tables 2 and 3. In figure 1, our present results are compared with the experimental results derived from literature. It is evident from the graphs that all sets of results are in reasonable agreement up to *Z* = 20. Beyond this point, the central potential results break down. This is because central potentials average results around the *l* = 0 values, for equivalent principal quantum numbers, leading to bigger deviations for higher *l* values. The nuclear charge *Z* amplifies such deviations. The symmetry-dependent potential, on the other hand, follows a pattern which is unique for every *l* value and for every atom. The symmetry-dependent results yield a reasonable prediction for the experimental values, but significant deviation from experiment persists even for this potential. It can be noticed that the influence of higher multipole corrections, beyond the lowest-order non-relativistic potential, is small.

In figure 2, our calculated results incorporating higher-order multipole potentials and finite nuclear mass corrections are compared with DFT calculations with LSDA and GGA exchange correlation functions [17] and with the experimental [26] results. Evidently, both sets of the DFT-XC spin polarized results compare well with experimental results, but our calculated results are also in reasonable agreement, and even better for some many-electron atoms like helium, lithium, beryllium, boron, neon, phosphorus and a few others. Our method is, nevertheless, not directly comparable with the DFT, making it a challenging task to explain the disparity between them.

**Figure 3.**
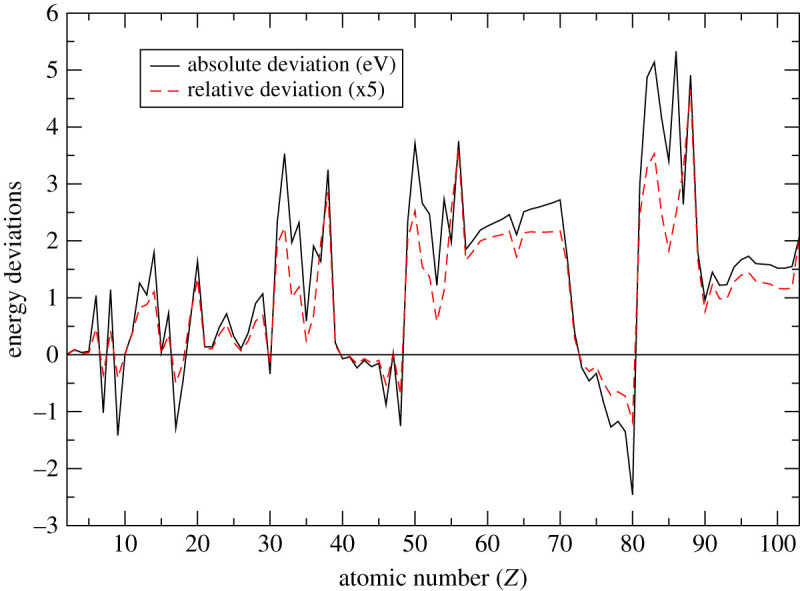
Ionization energies deviations, that is the absolute (Abs. Dev. = [*V*_fnm_ − Exp] in eV) and relative deviations, from the experimental results for the multi-electron atoms as a function of atomic number (*Z*). The relative deviations, multiplied by a factor of 5, are obtained by dividing the absolute deviation with the experimental ionization energies. Our higher-order multipole potential results with finite nuclear mass corrections are considered to be more realistic for the calculations of the deviations.

Figure 3 shows the absolute and relative energy deviations of our results from the experimental values. The absolute energy deviation is obtained by subtracting the experimental results from our higher-order multipole potential (*V*_fnm_) results with finite nuclear mass corrections. The relative energy deviations, on the other hand, are obtained by dividing the absolute deviations with the experimental values. Our relative deviations have been multiplied by a factor of 5 to fit on the same scale with the absolute deviation. Positive deviation implies that our binding energies for the respective orbitals are lower than the experimental values while negative deviation implies that our binding energies are higher. From the figure, it can be observed that the deviations show a high degree of correlation between them. It is also apparent that both deviations increase with atomic number (*Z*) to some extent. In general, the disparities between our present results, the experimental results, and both sets of DFT results point to the need for further research incorporating relativistic and other higher-order corrections in order to clarify the uncertainties existing in both theory and experiment.

## 4. Conclusion

We have generalized the symmetry-dependent all-electron potential for *n*-electron atoms. With this potential, all atoms are treated in the same way regardless of whether they are open- or closed-shell using only their system specific information. The scaling fractions (*m*/*n*) used have been derived for up to seven-electron systems and extended empirically using symmetry arguments for the rest of the *n*-electron systems [25]. In this study, the permanent (symmetric form of Slater determinants) expansion of single-electron spin-orbitals is used to express the wave function. This may be contrary to the fundamental postulates of quantum mechanics but, in a way, similar to the spatial expansion of the singlet states of helium atom using the Slater determinants. Our motivation for using such an expansion of the wave function was necessitated by the separability of the interaction potential leading to a form of a Hamiltonian for non-interacting fermions. The performance of the potential is tested against our previously developed central potential, and the literature DFT-XC, calculations in reproducing benchmark experimental results. The contributions of the lowest-order and higher-order non-relativistic interaction potentials in the various ionization energies are evaluated. The potential yields reliable ground-state ionization energies relative to the literature data. The major advantage of the derived potential is that it leads to a completely separable Hamiltonian for the many-electron atoms. This eliminates the need for self-consistent field iterations usually employed in other commonly used theoretical methods like the Hartree–Fock, CI and DFT. It hinges further on the premise that any two interacting electrons can exchange their relative position coordinates without exchanging their spin degrees of freedom. The suggested potential has a possibility for further improvement by incorporating the relativistic and other higher-order interactions.

## Data Availability

All data are embedded in the paper and in the supplementary material provided [28].

## References

[1] Hugenholtz NM. 1965 Quantum theory of many-body systems. Rep. Prog. Phys. **28**, 201-247. (10.1088/0034-4885/28/1/307)

[2] Verdebout S, Rynkun P, Jönsson P, Gaigalas G, Fischer CF, Godefroid M. 2013 A partitioned correlation function interaction approach for describing electron correlation in atoms. J. Phys. B: At. Mol. Opt. Phys. **46**, 085003. (10.1088/0953-4075/46/8/085003)

[3] Tobocman W. 1957 Many-body perturbation theory. Phys. Rev. **107**, 203-208. (10.1103/PhysRev.107.203)

[4] Cremer D. 2013 From configuration interaction to coupled cluster theory: the quadratic configuration interaction approach. Wiley Interdiscipl. Rev.: Comput. Mol. Sci. **3**, 482-503. (10.1002/wcms.1131)

[5] Kohn W, Sham LJ. 1965 Self-consistent equations including exchange and correlation effects. Phys. Rev. **140**, A1133-A1138. (10.1103/PhysRev.140.A1133)

[6] Cramer J. 2002 Essentials of computational chemistry. Chichester, UK: John Wiley and Sons, Inc.

[7] Hylleraas EA. 1929 Neue berechnung der energie des heliums im grundzustande, sowie des tiefsten terms von ortho-helium. Z. Phys. **54**, 347-366. (10.1007/BF01375457)

[8] Cohen ML. 1984 Application of the pseudopotential model to solids. Ann. Rev. Mater. Sci. **14**, 119-144. (10.1146/annurev.ms.14.080184.001003)

[9] Hellmann JJ. 1935 A new approximation method in the problem of many electrons. J. Chem. Phys. **3**, 61. (10.1063/1.1749559)

[10] Callaway J, Laghos PS. 1969 Application of the pseudopotential method to atomic scattering. Phys. Rev. **187**, 192. (10.1103/PhysRev.187.192)

[11] Jobunga EO. 2018 Parameter-free separable lowest-order non-relativistic Hamiltonian for helium atom. (https://arxiv.org/abs/1804.10058).

[12] Jobunga EO. 2020 Partitioning of the electron-electron interaction. New Horiz. Math. Phys. **4**, 23-28. (10.22606/nhmp.2020.42001)

[13] Jobunga EO. 2020 Pseudopotential for many-electron atoms. J. Adv. Appl. Phys. **2**, 20. (10.22606/jaap.2020.22002)

[14] Jobunga EO. 2020 Symmetry dependent analytical all-electron potential for helium atom. Res. Gate. Preprint. (10.13140/RG.2.2.26378.82884/4)

[15] Jobunga EO, Okeyo SO. 2020 Multipole expansion of integral powers of cosine theta. Sci. Rep. **10**, 20126. (10.1038/s41598-020-77234-4) 33208854 PMC7674459

[16] Kotochigova S, Levine ZH, Shirley EL, Stiles MD, Clark CW. 1997 Local-density-functional calculations of the energy of atoms. Phys. Rev. A **55**, 191-199. (10.1103/PhysRevA.55.191)

[17] Kraisler E, Makov G, Kelson I. 2010 Ensemble *v*-representable *ab initio* density-functional calculation of energy and spin in atoms: a test of exchange-correlation approximations. Phys. Rev. A **82**, 042516. (10.1103/PhysRevA.82.042516)

[18] Argaman U, Makov G, Kraisler E. 2013 Higher ionization energies of atoms in density-functional theory. Phys. Rev. A **88**, 042504. (10.1103/PhysRevA.88.042504)

[19] Klopper W, Bachorz RA, Tew DP, Hättig C. 2010 Sub-meV accuracy in first-principles computations of the ionization potentials and electron affinities of the atoms H to Ne. Phys. Rev. A **81**, 022503. (10.1103/PhysRevA.81.022503)

[20] Jobunga EO. 2017 Alternative multipole expansion of the electron correlation term. (https://arxiv.org/abs/1704.02009).

[21] Slater JC. 1928 Central fields and Rydberg formulas in wave mechanics. Phys. Rev. **31**, 333-343. (10.1103/PhysRev.31.333)

[22] Slater JC. 1930 Note on Hartree’s method. Phys. Rev. **35**, 210-211. (10.1103/PhysRev.35.210.2)

[23] Slater JC. 1930 Atomic shielding constants. Phys. Rev. **36**, 57-64. (10.1103/PhysRev.36.57)

[24] Zener C. 1930 Analytic atomic wave functions. Phys. Rev. **36**, 51-56. (10.1103/PhysRev.36.51)

[25] Jobunga EO. 2021 Determination of non-vanishing transition matrix elements for the many-electron systems. Res. Gate. (10.13140/RG.2.2.17329.30560/1)

[26] NIST. 2020 See http://www.physics.nist.gov/PhysRefData/Handbook/Tables.

[27] Koopmans T. 1934 Über die zuordnung von wellenfunktionen und eigenwerten zu den einzelnen elektronen eines atoms. Physica **1**, 104-113. (10.1016/S0031-8914(34)90011-2)

[28] Jobunga EO. 2022 Near-exact non-relativistic ionisation energies for many-electron atoms. Figshare. 10.1098/rsos.211779 PMC8941401 35345433

